# Role of Host Immune Response and Viral Load in the Differential Outcome of Pandemic H1N1 (2009) Influenza Virus Infection in Indian Patients

**DOI:** 10.1371/journal.pone.0013099

**Published:** 2010-10-01

**Authors:** Vidya A. Arankalle, Kavita S. Lole, Ravi P. Arya, Anuradha S. Tripathy, Ashwini Y. Ramdasi, Mandeep S. Chadha, Shashi A. Sangle, Deelip B. Kadam

**Affiliations:** 1 National Institute of Virology, Pune, India; 2 Sassoon General Hospital, Pune, India; University of California Los Angeles, United States of America

## Abstract

**Background:**

An unusually high number of severe pneumonia cases with considerable mortality is being observed with the pandemic H1N1 2009 virus infections globally. In India, all mild as well as critically ill cases were admitted and treated in the government hospitals during the initial phase of the pandemic. The present study was undertaken during this early phase of the pandemic.

**Methodology:**

The role of viral load and host factors in the pathogenesis were assessed by examining 26 mild (MP), 15 critically ill patients (CIP) and 20 healthy controls from Pune, India. Sequential blood and lung aspirate samples were collected from CIP. Viral load and cytokines/chemokine levels were determined from the plasma and lung aspirates of the patients. TLR levels were determined by staining and FACS analysis. Gene profiling was done for both cells in the lung aspirates and PBMCs using TaqMan Low Density arrays. Antibody titres and isotyping was done using HA protein based ELISAs.

**Principal Findings:**

13/15 critically ill patients expired. All plasma samples were negative for the virus irrespective of the patient's category. Sequential lung samples from CIP showed lower viral loads questioning association of viral replication with the severity. Anti-rpH1N1-09-HA-IgG titres were significantly higher in critically ill patients and both categories circulated exclusively IgG1 isotype. Critically ill patients exhibited increase in TLR-3, 4, 7 and decrease in TLR-2 expressions. The disease severity correlated with increased plasma levels of IL1RA, IL2, IL6, CCL3, CCL4 and IL10. Majority of the immune-function genes were down-regulated in the PBMCs and up-regulated in the cells from lung aspirates of critically ill patients. No distinct pattern differentiating fatal and surviving patients was observed when sequential samples were examined for various parameters.

**Conclusions:**

Disease severity was associated with pronounced impairment of host immune response.

## Introduction

The first pandemic of this century was unexpectedly caused by a novel swine-origin H1N1 virus, the pandemic H1N1 (2009) virus (p-H1N1-09). Mexico was the first country to be affected in early March with reports of mild respiratory infection as well as severe pneumonia cases and considerable mortality [1], [2]. Several countries were subsequently affected reporting variable mortality, smoking, pregnancy and obesity being important risk factors for severe disease [3], [4], [5], [6].

On 1^st^ August 2009, a 14 year-old girl without history of known risk factors succumbed to p-H1N1-09 infection in Pune, western India representing the first fatality from the country. As of 21^st^ April 2010, India has reported 1483 deaths during the pandemic (http://pib.nic.in/h1n1/h1n1.asp), Pune contributing to 173 cases (http://www.maha-arogya.gov.in/march-april%202010.htm).

During the initial phase of the pandemic, designated wards in the government hospitals admitted every mild case, treated with Oseltamivir and discharged after recovery. A special intensive care unit treated the critically ill patients. The present study was undertaken during this very early phase of the pandemic. To understand the basis of differential disease presentation/outcome, we investigated 26 mild cases and 15 critically ill patients during 1^st^ August – 19^th^ September 2009. This report provides comparative data on viral load, cytokines, gene-profiling, Toll-like-receptor (TLR) levels, antibody titres and antibody isotypes.

## Materials and Methods

### Patients and clinical specimens

Ethical clearance for the study was obtained from, ‘Institutional Human Ethical Committee’ as part of the pandemic influenza investigations. Written consent was obtained from all the participants involved in the study. For minors and critically ill patients it was obtained from parent/guardian. Patients confirmed to have p-H1N1-09 infection by a positive real time PCR test (http://www.who.int/csr/resources/publications/swineflu/CDCRealtimeRTPCRprotocol_SwineH1Ass-2009_20090428) were studied. These included 15 patients admitted to Intensive Care Unit and requiring mechanical ventilator support and 26 suffering from mild respiratory symptoms. All the mild cases were ambulatory patients admitted to a designated hospital. In the initial phases of the pandemic during which this study was performed, patients suggestive of Influenza-like illness were admitted to designated ward of a Corporation hospital. Throat swabs were collected and sent to the National Institute of Virology for diagnosis. Osletamivir treatment was initiated immediately after the confirmation of diagnosis. These patients were discharged after the completion of the treatment. On the contrary, majority of the severe patients were admitted only after the development of serious respiratory consequences. Same protocol was followed for diagnosis and antiviral treatment.

A single blood sample was collected from mild cases, 1-3 days after the development of respiratory symptoms. As controls, blood samples from 20 apparently healthy individuals were collected. Sequential blood/lung aspirate samples (standardized tracheal aspirates) were collected from the critically ill patients. The first sample was collected within 3 days for 13 patients; while one each was collected on 4 and 8 (pregnant woman) days after the appearance of symptoms (Table 1). Blood and lung aspirates were transported to the lab within half an hour of collection. Lung aspirates obtained from severe cases were immediately aliquoted and frozen at −80°C. An aliquot was mixed in 1∶3 proportions with the RNAlater and stored at −80°C for gene analysis. Hundred microlitre of the blood sample from every patient was processed immediately for TLR staining as described below. In parallel, blood samples were immediately processed for the isolation of Peripheral Blood Mononuclear Cells (PBMCs) by density gradient centrifugation using Ficoll-Hypaque (Sigma). Plasma layer was removed and stored at −80°C in aliquots; cell pellets were stored in 500 µl RNALater solution (Ambion) at −80°C.

**Table 1:** Characteristics of the critically ill patients admitted to the Intensive care unit.

Identification	Age/sex	Sickness duration before admission (days in hospital)	Lung radiological findings (of 6 zone involvement)	Co-morbidities	Outcome	Lung aspirates
p-09-S1	28M	3 (7)	<3	Asthma	Survived	Yes
p-09-S2	19M	3 (4)	<3	None	Survived	Yes
p-09-S3	25F	8 (10)	≥3	Pregnancy, 16 wks	Death	Yes
p-09-S4	53M	1 (8)	≥3	RHD	Death	Yes
p-09-S5	40M	3 (10)	≥3	None	Death	Yes
p-09-S6	40M	3 (12)	≥3	DM/HT	Death	Yes
p-09-S7	32F	3 (4)	<3	None	Death	Yes
p-09-S8	35F	2 (2)	≥3	RHD	Death	Yes
p-09-S9	46M	4 (10)	≥3	HIV-1 +ve	Death	No
p-09-S10	35F	1 (1)	≥3	None	Death	No
p-09-S11	22F	2 (2)	≥3	None	Death	Yes
p-09-S12	14F	1 (1)	≥3	None	Death	Yes
p-09-S13	40F	2 (1)	≥3	RHD	Death	No
p-09-S14	13F	2 (1)	≥3	None	Death	No
p-09-S15	43F	1 (2)	≥3	Obesity	Death	No
s-09-S21**	36F	3 (39)	≥3	None	survived	Yes

**Suffering from seasonal influenza; RHD =  Rheumatoid heart disease, DM =  Diabetes mellitus, HT =  Hypertension.

### ELISA for IgG-anti-p-H1N1 antibodies and isotyping

A highly sensitive and specific ELISA was carried out by coating the wells with purified recombinant HA protein (expressed in baculovirus system) of p-H1N1-09 virus, sera at 1∶100 dilution and anti-human-IgG-HRP conjugate as the detector antibody [7]. Isotyping was done as described earlier [8].

### TLR staining and flow cytometry analysis

Anti-human antibodies for TLR 2, 3, 4 and 9 (eBioscience, USA) and TLR 7 and TLR 8 (Imgenex, USA) were used for the staining. For surface staining of TLR 2 and 4, 100 µl of whole blood was lysed with BD FACS lysis solution (BD Biosciences, USA), washed, fixed and processed for staining with anti-human TLR 2 (FITC conjugated) and TLR 4 (PE conjugated) antibodies respectively. For intracellular staining, 100 µl of the whole blood was fully lysed with BD FACS lysing solution (Becton Dickinson), washed twice with Perm-wash buffer (BD bioscience) and processed for staining with the following anti-human antibodies: PE- TLR 3, FITC- TLR 7, FITC- TLR8 and PE-TLR 9 respectively. Stained cells were resuspended in 500 µl 1% paraformaldehyde, analysed on FACScalibur flow cytometer (Becton Dickinson).On the basis of forward and side scatter plot lymphocytes and monocytes were gated and data analysis was done using BD FACSDivasoftware.TLR levels were expressed as median fluorescence intensity (MFI).

### Cytokine measurements

Concentrations in the plasma/lung aspirates were determined for seventeen cytokines/chemokines (IL1α, IL1β, IL6, TNFα, IL17, IL1RA, IL2RA, IL10, IL2, IL12p40, IL12p70, IFNγ, MIF, TRAIL, CXCL8, CCL3 and CCL4) on Bio-Plex Protein Array System (Bio-Rad, Hercules, CA, USA) using Milliplex Map Kit (Millipore) as per the manufacturer's instructions.

### Total RNA extraction and gene expression analysis

Frozen lung aspirates were thawed, centrifuged to pellet down the cells and pellets were used to isolate RNA. Total RNA was extracted from PBMCs and lung aspirate cells by using Ribopure Kit (Ambion) as per the manufacturer's instructions. RNA was eluted in 100 µl elution buffer, quantitated using Nanodrop (ND-1000) and processed for quality check in Agilent bioanalyzer (Agilent, U.S.A). Equal quantities of RNA (500 ng) with ≥7 RIN value were processed further for cDNA synthesis using High Capacity cDNA Reverse Transcription Kit (Applied Biosystems, U.K.). All cDNAs were tested in real time PCR assay using TaqMan primers and probe for 18S rRNA (Applied Biosystems, U.K.) to ensure efficient cDNA synthesis. cDNAs were mixed with equal volumes of TaqMan 2X PCR master mix from one-step RT-PCR kit (Applied Biosystems) and 125 ng (RNA equivalent) cDNAs were loaded per port of the TaqMan Low Density Array card (TLDA) of the Human Immune panel (Applied Biosystems, U.K.) and run on 7900HT Fast Real-Time PCR system (Applied Biosystems, U.K.). Relative gene expression values were obtained employing comparative Ct method using Applied Biosystems' Relative Quantification (RQ) Manager Software v1.2. cDNAs from six healthy individuals processed similarly were considered as calibrators. 18s RNA was used as an endogenous control. Relative quantitation values of each study group were used to calculate mean RQ values. For cluster analysis, relative quantitation values were log2 transformed and hierarchically clustered with analysis software (Cluster 3.0) [9].

### Viral RNA Titres

Viral RNA was extracted from 140 µl plasma/lung aspirates using QIAamp Viral RNA Mini Kit (Qiagen, Germany) as per the manufacturers' instructions. For viral RNA quantitation, CDC primers and probe were used. The target HA gene from an Indian isolate was cloned to obtain in vitro transcripts, serial 10-fold dilutions of the RNA were used to generate a reference curve. A linear relationship was obtained from 10^10^–10^2^ starting copies/reaction (r^2^ = 0.99), the detection limit being 100 copies.

### Statistical Analysis

Levels of cytokines and chemokines were analyzed after log transformation and a value of 0.2 pg/ml was used in the case of undetectable concentration of cytokine or chemokine in the tested samples. The Mann-Whitney U or Fisher exact tests were used for group comparisons of numerical and categorical data respectively. For all analyses, a P value of less than 0.05 derived from a two tailed test was considered significant. All statistical analyses were performed with ‘SPSS11.0 for Windows’ software (SPSS Inc.).

## Results

All the 26 mild cases (Male: Female ratio 12∶14, age range 6–51 yrs) had fever/history of fever in last 3 days. The other symptoms included sore throat/cough (14/26), nasal discharge (6/24), headache/bodyache (5/26), diarrhea (2/26) and breathlessness (4/26). Radiological examination was not indicated and hence not performed. Nasal oxygen was not required.

Of the 15 critically ill patients, 2 survived (Table 1). The male:female ratio was 6∶9, the age range being 13–53 years. Associated co-morbidities were present in 1/2 surviving and 7/13 fatal patients. Importantly, radiological findings showed that all the fatal cases had ≥3/6 lung zonal involvement and required ventilator assistance. Both the surviving patients initially needed nasal oxygen but were put on ventilator subsequently. The patients were either treated with Osletamivir alone (n = 7) or in combination with Zanamivir (n = 8). From the endotracheal tubes of 4 patients Acenotobacter was isolated. None of the 26 mild cases had recognizable risk factors. S21, a 36 year female suffering from severe seasonal influenza survived after 39 days of hospitalization.

### Viral load

**Figure 1:**
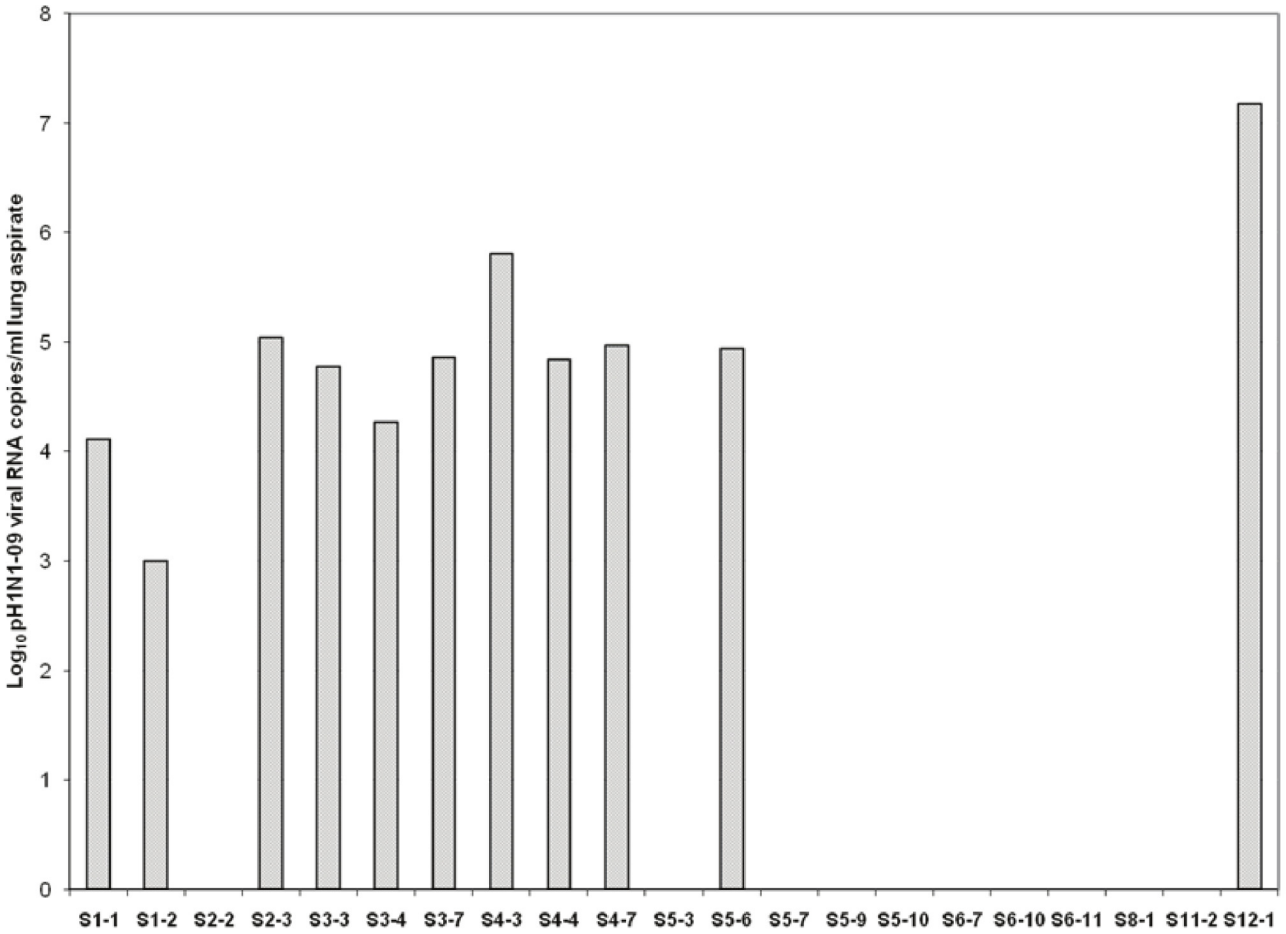
Virus titres in the lung aspirates of critically ill patients. RNA log_10_ copies/ml were determined by HA gene based real-time PCR assay. Y-axis represents different patients and samples obtained days post admission of the sequential samples of respective patients (S1-1, S1-2, …and S12-1).

Plasma samples from both patient categories were negative for pH1N1-09 influenza virus RNA. Sequential lung aspirate samples of 6 patients (2–5 samples) demonstrated gradual/continued lower or no viral load. Of the single lung aspirate from 3 patients with rapid death, two were negative for viral RNA while one exhibited 1.51×10^5^ copies/ml (Figure 1).

### Antibody titres

Serum samples available from 4 critically ill patients exhibited HI titres of 40–320. At admission, the geometric mean titres (GMT) of anti-rpH1N1-09-HA-IgG antibodies (ELISA) were significantly lower in the mild infections (800, 95% CI values 571–1120) than the critically ill patients (4996, 95% CI values 3970–6288) (p<0.0001). The last blood sample collected 4–11 days later from 11 critically ill patients showed >4 fold rise in antibody titres. IgG1 was the exclusive isotype in both the categories.

### TLR levels

**Figure 2:**
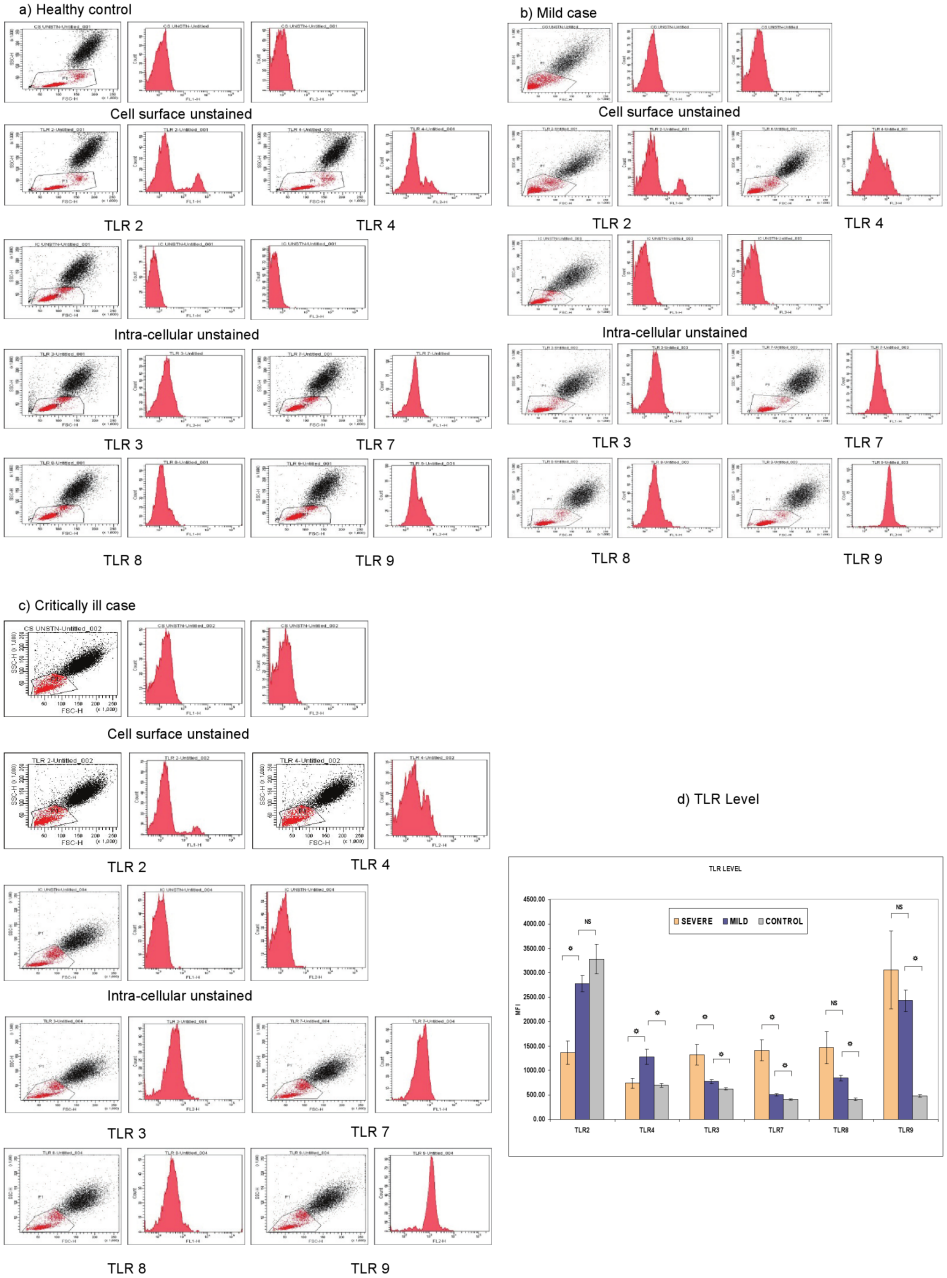
TLR levels in the blood lymphocytes and monocytes. TLR levels of peripheral blood from the patients and healthy controls were determined separately by either surface staining or intracellular staining of the cells using TLR specific antibodies followed by FACS analysis. **Scatter dot plots and histograms:** A representative sample each from different categories, **a**) healthy individual, **b**) mild case, **c**) critically ill case**, d**) **TLR levels:** Expressed as median fluorescence intensities (MFI) for different categories of patients.

Figure 2a shows representative FACS plots of TLR analysis. The expression of TLR3, TLR4, TLR7, TLR8 and TLR9 was significantly higher in mild cases than controls (p = 0.0081, 0.0000, 0.0065, 0.0000, 0.0000 respectively), TLR2 not being different (p = 0.22) (Figure 2b). Comparison of the disease categories revealed significant decrease in TLR 2 (p = 0.0009) and rise in TLR3, TLR4, TLR7 levels (p = 0.0078, 0.019, 0.0070 respectively) in critically ill patients. Levels of TLR8 and TLR9 remained unchanged (p = 0.12, 0.93).

### Cytokine levels

Comparison among healthy controls and disease categories showed no difference in the levels of IFNγ, IL12p70 and IL2RA were significantly lower in mild cases and higher in critically ill patients (Table 2). All other molecules showed significantly higher levels in both patient categories. The overall pattern reflects pandemic-H1N1-09 influenza infection. The disease severity correlated with significant increase in IL1RA, IL2, IL6, CCL3, CCL4 and IL10 levels in critically ill patients.

**Table 2:** Levels of chemokines and cytokines in the peripheral blood.

Values represent Median log_10_pg/ml (range)							
	Critically ill patients	p Valuea	p Valueb	Mild cases	p Valuec	Healthy Controls	Lung Aspirates
**Pro-Inflammatory Cytokines**							
IL1α	1.64 (−0.70–3.26)	0.2061	0.0032	−0.70 (−0.70–3.26)	0.0312	−0.70 (−0.70–3.28)	2.81 (0.48–3.21)
IL1β	0.48 (−0.70–1.05)	0.2639	0.0053	0.32 (−0.70–1.80)	0.0006	−0.70 (−0.70–0.72)	2.05 (0.48–2.68)
IL6	2.14 (0.40–3.11)	0.0022	0.0000	0.32 (−0.70–3.11)	0.0001	Undetected	2.59 (0.48–3.29)
TNFα	0.48 (0.48–1.06)	0.3697	0.0000	0.70 (−0.70–4.41)	0.0000	Undetected	0.48 (0.48–0.48)
IL17	0.48 (−0.70–2.14)	0.2608	0.0002	0.18 (0.08–1.62)	0.0000	−0.70 (−0.70–0.61)	0.48 (0.48–1.07)
**Anti-Inflammatory Cytokines**							
IL1RA	1.23 (−0.70–3.20)	0.0220	0.0000	0.82 (−0.70–1.84)	0.0001	Undetected	2.55 (0.48–3.01)
IL2RA	0.91 (−0.01–1.18)	0.4438	0.0000	−0.10 (−0.10–2.92)	0.0000	Undetected	2.27 (0.48–2.58)
IL10	1.71 (−0.70–2.59)	0.0135	0.0000	−0.70 −0.70–2.38)	0.0011	Undetected	0.48 (0.48–2.59)
**Th-1 Cytokines**							
IL2	0.48 (0.01–1.31)	0.0002	0.0000	−0.70 (−0.70–0.53)	0.0032	Undetected	0.48 (0.48–2.52)
IL12p70	0.26 (−0.70–0.32)	0.6705	1.0000	0.20 (0.05–0.32)	0.4417	0.20 (0.05–0.32)	0.48 (0.48–2.74)
IL12p40	0.48 (−0.70–2.76)	0.2655	0.0000	0.32 (0.08–2.18)	0.0000	Undetected	2.40 (0.48–2.74)
**Chemokines**							
CXCL5	3.30 (−0.70–3.71)	0.1580	0.0001	−0.70 (−0.70–3.55)	0.0039	Undetected	2.92 (1.13–3.78)
CXCL8	1.37 (−0.70–3.63)	0.5150	0.0000	1.10 (0.20–3.65)	0.0000	Undetected	4 (0.48–4.04)
MIF	3.33 (3.13–4.24)	0.4368	0.0072	3.45 (3.13–4.05)	0.0009	3.08 (2.11–3.69)	4.18 (1.08–5.42)
CCL3	1.66 (−0.70–2.25)	0.0224	0.0000	−0.70 (−0.70–2.28)	0.0032	Undetected	1.54 (0.48–2.80)
CCL4	1.89 (−0.70–2.84)	0.0495	0.0000	1.57 (−0.70–2.0)	0.0001	Undetected	2.59 (0.08–3.43)
TRAIL	0.15 (−0.70–3.09)	0.2866	0.0032	0.15 (−0.70–3.09)	0.0012	−0.70 (−0.70–3.51)	1.89 (−0.30–2.76)

a* *p* value between critically ill patients and mild cases.

b* *p* values between critically ill patients and healthy controls.

c* *p* values between mild cases and healthy controls.

### Gene-profiling (PBMCs)

Analysis of gene expression profiles revealed significant findings; please see Table S1:

#### Comparison of controls with disease categories

1. Down-regulation of IL15, NOS2A, CCR4 and up-regulation of IL17, CYP7A1, SELE, IL5, RPL3L genes in both patient categories indicative of response to p-H1N1-09 virus infection.
2. Only in critically ill patients, CCL3, CCL19, CCR7, IL1A, 1L1B, IFNG, CD34, CD80, BAX, COL4A5, EDN1, TNFRSF18, PTPRC, SKI were down-regulated while HLA DRB1, IL9 and IL6 were up-regulated.
3. CCL2 and CXCL11 were up-regulated only in mild cases while IL-4 was up-regulated in critically ill patients and down-regulated in mild cases.

#### Comparison among the patient categories

A large number of genes were significantly down-regulated in critically ill patients than in the mild cases (CCL2, CCR5, TNF, IL2, IL13, CD3E, CD4, CD8A, CD28, CD40, CD40LG, CD86, CTLA4, FASLG, HLA DRA, ICOS, ACE, BCL2, C3, CSF1, GNLY, GZMB, IKBKB, LTA, PRF1, SMAD3, SMAD7, and TBX21).

### Lung aspirate analysis

Tables 2 and S1 represent the status of cytokines and chemokines at protein and gene levels respectively in the lung aspirates/cells of lung aspirates of critically ill patients. In the absence of similar samples from mild cases/healthy controls for ethical reasons, no comparison was possible.

Comparison of plasma and lung aspirates of critically ill patients identified higher levels of CXCL8 and IL12p70 in the lung aspirates. No significant difference was noted for the other molecules. None of the plasma markers of severity exhibited higher levels in the lung aspirates.

Following patterns emerged from the gene profiling comparisons of PBMCs and lung cells of critically ill patients:

1. Genes showing down-regulation in both: CD40LG and SMAD7. Both genes were significantly down-regulated in the PBMCs of critically ill patients than mild cases.
2. Genes showing up-regulation in both: CCL2, IL6, IL10, IL17, HLA DRB1, CYP7A1, SELE. Except CCL2, all other genes exhibited insignificant difference when PBMCs from mild and critically ill patients were compared.
3. Genes showing up-regulation in lung and down-regulation in PBMCs: CCL3, CCL19, IL1A, IL1B, TNF, CD80, ACE, C3, COL4A5, CSF2, EDN1, NOS2A.
4. Genes showing basal levels in PBMCs and up/down-regulation in the lung: Except for down-regulation of IL12A, all other genes were up-regulated in lung. These included: CXCL8, CXCL10, CXCL11, ICAM1, CSF1, HMOX1, STAT3, CD68, CSF3, FN1, NFKB2, TFRC, VEGF and IL12B.
5. Genes showing basal levels in the lung and up/down regulation in PBMCs: Except for the up-regulation of IL5 and IL9, all other genes were down-regulated in PBMCs (IFNG, CCR4, CCR7, CD3E, CD4, CD8A, CD28, CD34, CTLA4, BAX, BCL2, GNLY, IKBKB, PRF1, PTPRC, SKI, SMAD3, TBX2 and TNFRSF18).
6. Genes unchanged in lungs and PBMCs: CD40, CD86, FASLG, GZMB, LTA, CCR5 and ICOS.

**Figure 3:**
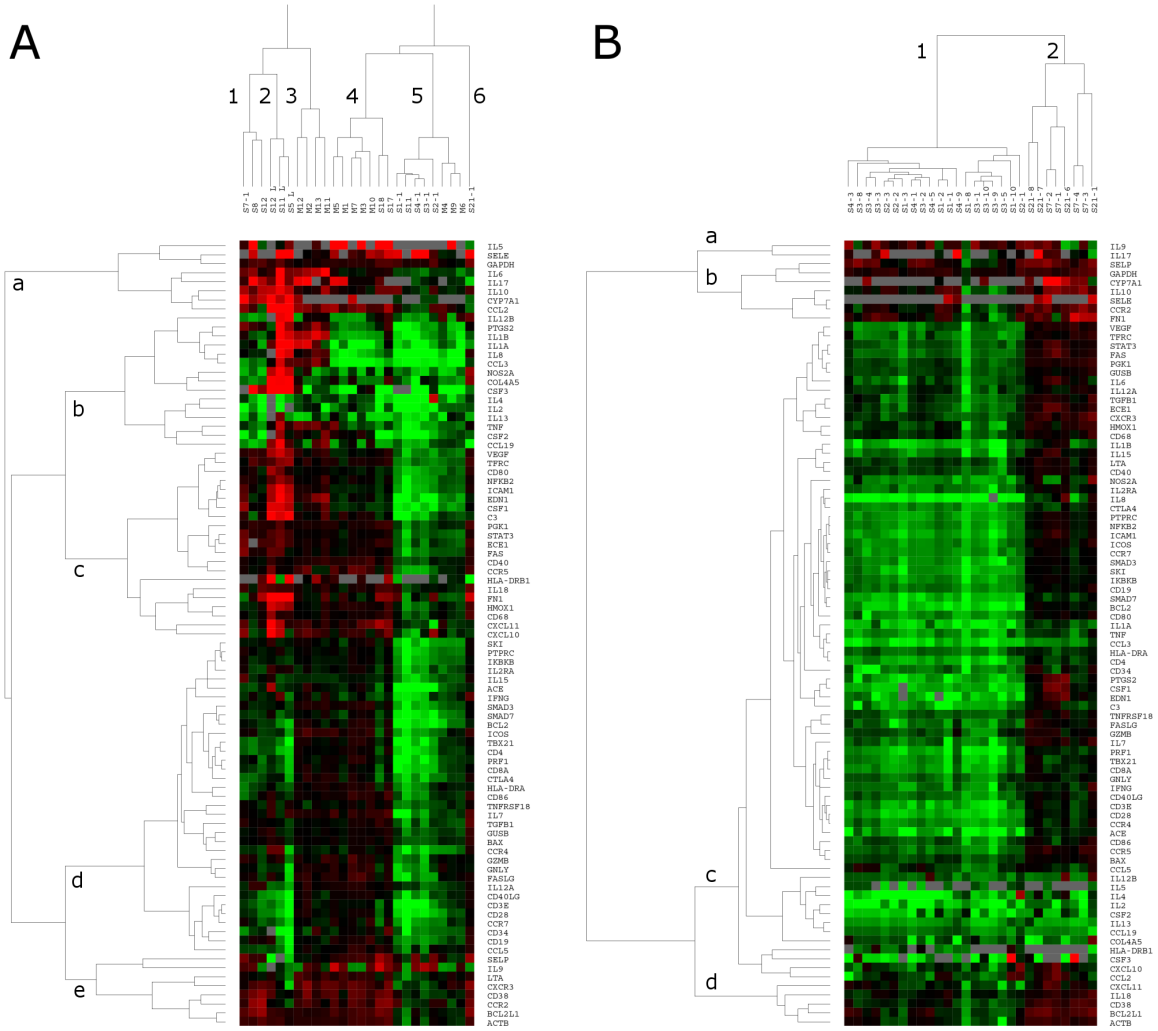
Gene expression analysis. Gene expression profiles of total PBMCs from blood samples of mild and critically ill cases and lung aspirate cells from critically ill cases were determined using TaqMan Low Density immune panel arrays. PBMCs from six healthy individuals were taken as controls, which were treated as replicate arrays to calculate the mean baseline expression level for each gene. The fold changes in the gene expression levels were calculated in relation with the controls. Values for eighty-seven genes were hierarchically clustered on log2 transformation. The corresponding gene of each cluster is listed by a gene symbol on the right-hand side of the images. **a**) **Cluster image of gene expression analysis of PBMCs from mild and critically ill cases and lung aspirate cells from critically ill cases:** PBMC samples from mild cases are denoted as M (M1, M2, M13). First sample taken after admission from each critically ill case was taken for the comparison and are denoted as S1-1, S2-1, S21-1. Single samples from critically ill patients are denoted as S8, S12 etc. Lung aspirate samples are denoted as L. **b**) **Cluster image of gene expression analysis of sequential blood samples from critically ill cases:** Total PBMCs from the sequential blood samples of severe cases obtained on different post admission days are denoted as S1-1, S1-2, …S21-8).

Expression patterns of genes were also analyzed by hierarchical clustering. Results are graphically represented by assigning a specific color to each cell on the basis of the expression levels. Genes showing no changes in expression levels (as compared to controls) are shown as black. Upregulated genes are shown in red with increasing intensities in proportion to the expression levels and in different intensities of green indicating levels of downregulation. Figure 3a shows cluster image of samples from mild and critically ill cases taken on admission. Seasonal flu sample (S21) showed a distinct gene expression pattern and formed a separate cluster (cluster 6) from all other samples. Three lung samples from critically ill patients also formed a separate cluster as most of the proinflammatory genes were upregulated (cluster 2). Remaining samples formed mixed clusters (cluster 4 and 5) which included both mild and critically ill patients. Overall, no distinct clustering pattern for the patients was observed for the severe and mild cases. With respect to gene clustering, clusters A and B contained genes associated with inflammation.

Comparisons of genes of lungs and PBMCs of the same 3 patients or all the critically ill patients as a group yielded similar results.

Comparison of lung cytokines and chemokines at protein and gene levels showed comparable elevated levels of IL12B, CXCL8, IL1B, CCL3, IL1A and IL6 while IL17, TNF and IL10 were elevated only at the gene level. IFNG was at basal level both at gene and protein levels.

### Sequential analysis

Variable numbers of sequential blood samples were available from critically ill patients. Comparisons of TLRs did not show any distinguishable pattern (data not shown). For evaluating gene expressions, a separate cluster analysis was carried out for the sequential PBMC samples of critically ill patients (Figure 3b). Samples formed two distinct clusters. Samples from S1, S2, S3 and S4 formed a single cluster (cluster 1) showing significant downregulation of most of the immune function genes. Cluster 2 contained all sequential samples from the seasonal flu case (S21) and one pH1N1 case (S7). Though most of the gene expression levels were similar to survived case, S7 did not survive. Genes formed four clusters, A, B, C and D. Cluster C contained majority of the analyzed genes (73/87) and all were downregulated in the cluster 1.

**Figure 4:**
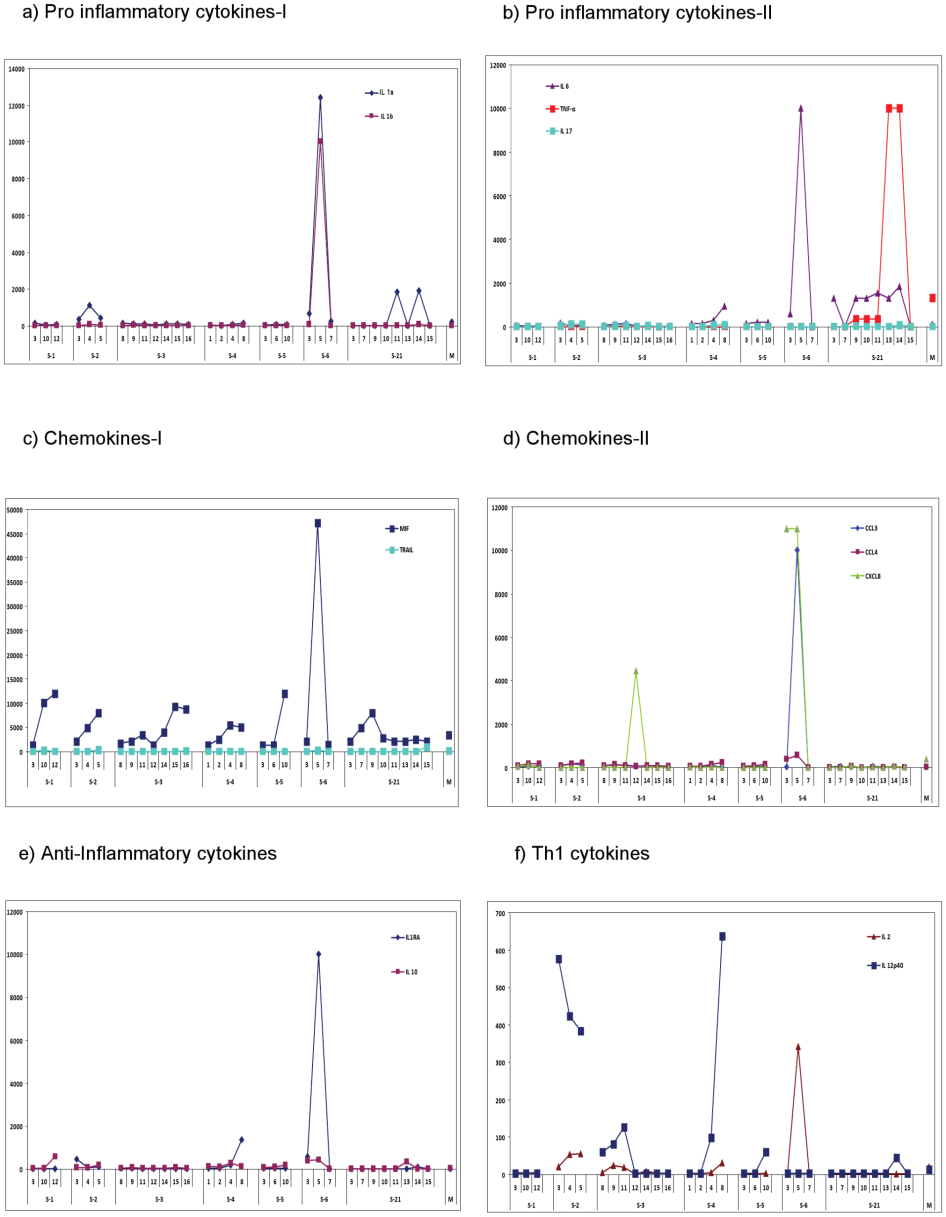
Chemokine and cytokine levels in plasma. The concentrations of cytokines and chemokines were determined using Milliplex Map Kit in the sequential blood samples obtained on different post infection days from critically ill patients. M represents mean values of the corresponding molecule levels in the plasma of 21 mild cases. **a**) **Pro-inflammatory cytokines-1. b**) **Pro-inflammatory cytokines-2. c**) **Chemokines-1. d**) **Chemokines-2. e**) **Anti-inflammatory cytokines. f**) **Th1 cytokines.**

The dynamics of cytokines/chemokines at protein levels (6 patients) are shown in Figure 4. Data for the single critical patient suffering from seasonal influenza (S21) is also presented. Comparison of plasma cytokines and chemokines did not show any distinct pattern in the sequential samples of the fatal or survived cases. Patient S6 did exhibit different pattern when compared to others.

## Discussion

This first comprehensive study addresses important issues of the identification of markers for severity of p-H1N1-09 infection and dynamics of immune responses in severe disease by evaluating several parameters. The investigations were initiated during the early phase of the pandemic when isolation of all the p-H1N1-09 mild infections in designated wards was obligatory. Therefore, we could collect samples before the initiation of the antiviral therapy and the data truly represents acute phase of mild disease. On the other hand, critically ill patients were given Oseltamivir immediately after the confirmation of the infection and the samples were collected subsequently. As underlying medical conditions were present in one of the 2 survivors and 7/13 critically ill patients, association of co-morbidities alone does not seem to be responsible for complications or aberrant immune response.

The absence of viremia in both patient categories and relatively low viral load in the lung aspirates of the critically ill patients suggest that enhanced replication of the virus may not be an important contributor to the pathogenesis (Figure 1). The viral load in lung aspirates was independent of fatality. In contrast, among the Spanish patients [10], 93% and 57% of the mild and critical cases respectively were positive for serum viral RNA, with no significant difference in the viral load. Both studies used CDC primers for real time PCR (http://www.who.int/csr/resources/publications/swineflu/CDCRealtimeRTPCRprotocol_SwineH1Ass-2009_20090428) and the critical cases were bled when already on Oseltamivir treatment, negating sensitivity of the PCR, effect of antiviral therapy or delay in collection of samples to be responsible for different results. The absence of uniform mutations in the fatal cases-derived Indian p-H1N1-09 isolates suggests limited/no role of mutant virus in the pathogenesis [11]. Viremia was associated with the outcome of H5N1 infection, 9/11 fatal and 0/5 non-fatal cases being viremic [12].

Serum HI-antibody positivity was noted in 1/15mild, 2/10 critically ill Spanish patients and 4/4 critically ill patients from India. ELISA could detect antibodies in every patient. The absence of viremia among the Indian patients may be due to an early antibody response.

The presence of IgG-anti-p-H1N1-09 antibodies in all the severe Indian patients (HI antibodies in all the 4 screened) does indicate switch from the innate to adaptive immunity. Poor outcome despite the switch may probably be attributed to the timing of the shift or role of antibodies in disease severity. Significantly higher titres of IgG-anti-p-H1N1-09 antibodies in the critically ill patients supports the role in pathogenesis. This observation is in sharp contrast with that for the SARS patients examined from Canada [13] documenting significantly lower anti-SARS CoV spike antibody titres in the critically ill patients. Though the presence of neutralizing antibodies protect against Influenza virus infection, clearance of the infection is mediated by cellular immunity. The exclusive presence of IgG1 antibodies in both patient categories document Th2 bias with significant enhancement with severity. This observation correlates with the elevated levels of TLR3, 4, and 7, Th2 cytokines (IL6 and IL10) at protein level and IL4, IL5, IL6, IL10 at gene level.

We identified higher plasma levels of IL1RA, IL2, IL6, CCL3, CCL4 and IL10 as markers of severity. Of these, IL6 is a known marker of influenza disease severity with probable involvement in tissue damage [12], [14]. CCL3 and CCL4 are important mediators of virus induced inflammation. IL10 is recognized as a regulatory (anti-inflammatory) cytokine and can act on multiple cell types to regulate immune and inflammatory responses [15]. IL6 is known to be responsible for regulating plasma levels of IL1RA and IL10 [16]. Among Spanish patients, serum IL15, IL12p70 and IL6 were recognized as the hallmark of severity [10]. Significant increases in TLR levels without corresponding rise in cytokines suggest aberrant immune response in critically ill patients. The possibility of viral proteins diminishing cytokine production cannot be ruled out. Similar to *in-vitro* studies [17], [18], cytokine storm associated with pathogenesis of H5N1 infection [12] was not the feature of p-H1N1-09 infection. The observations of no increase in the levels of plasma IFNG in both patient categories when compared to controls as well as basal gene expression in the lungs of critically ill patients point out lack of co-ordination in the modulation of innate and adaptive immune responses.

At PBMC gene level, down-regulation of a large number of genes was associated with disease severity (Table S1, Figure 3b). This profile is indicative of massive infiltration of monocyte-recruited neutrophils, DCs/macrophages to the target organ for mounting immune response/tissue repair and/or viral protein-induced shut down of the cellular genes, as these cells are known to efficiently replicate the virus [19], [20]. Additional studies are required to examine if the virus specifically down regulates host antiviral genes or dictates generalized shut down of host mRNA synthesis [19], [21], especially in relation to the disease severity. Paradoxically, constant inflammatory signals provided by the significant increase in several chemokines (CCL2, CCL3, CCL19, CXCL8, CXCL10, CXCL11) and pro-inflammatory cytokines (IL6, IL17, IL1A, IL1B, TNF) in the lung cells may have resulted in massive infiltration of leucocytes and excessive tissue damage. This is to our knowledge the first report on p-H1N1-09-induced gene expression in human lung cells.

As against differential cytokine levels and viral load observed in a male and pregnant female fatal H5N1 case [21], except for higher down-regulation of IL1B/IL2, the pregnant woman (S3) in our series was similar to others. It was intriguing to note that despite attempts of mounting immune response similar to mild-recovered patients, the patient S7 did succumb to the disease. It is important to recognize that the current study examines virus-host interactions throughout the course of severe disease, limitation of the study being absence of similar data from mild cases.

We could investigate a critical case suffering from seasonal influenza (S21). As against similar patterns recorded for pandemic influenza cases, distinct differences were recorded for this case (Figure 3a, 3b).

In the ferret model with 27% mortality based on the >20% weight loss following infection with A/California/07/2009 pandemic influenza virus [22], sequential lung sampling documented decreased gene expression of CCL2, CXCL10, TNFA and IL1B on day 7 when animals show highest weight loss. Comparison of the human data presented in our study (Table S1) shows that in the lungs of the critically ill patients all these and several other chemokines/inflammatory cytokines are over-expressed. Thus, the gene expression profiles at the time of overwhelming symptoms in the lungs of the infected ferrets (decreased expression) do not match the data on severe human cases from the present series (elevated expressions).

While finalizing the manuscript, we came across two studies from Hong Kong [23], [24]. The mild patients (n = 22) were non viremic while 13% of the 23 fatal cases were viremic. Data from Spain, Hong Kong and India suggests the role of host genetics in immune response to the pandemic-causing virus.

In conclusion, our data confirms earlier findings of dysregulated host response in severe infections with H5N1 [12] and 1918 influenza viruses [25], [26], [27]. However, the mechanisms leading to similar end results seem to vary with the type of viral infection. In-depth studies to understand the role of virus/viral proteins in modulating host response need to be undertaken on priority. Identification of specific pathways might provide clues to include immunomodulators for the treatment of severe cases, albeit in conjunction with potent antivirals.

## Supporting Information

Table S1 Gene profiles of PBMCs and lung aspirate cells of patients.
(0.17 MB DOC)
