# Supplementary material for: Role of Host Immune Response and Viral Load in the Differential Outcome of Pandemic H1N1 (2009) Influenza Virus Infection in Indian Patients
Source: PLoS One. 2010 Oct 1;5(10):e13099. doi: 10.1371/journal.pone.0013099 (PMC2948498; doi:10.1371/journal.pone.0013099)
Supplement: Table S1 — Gene profiles of PBMCs and lung aspirate cells of patients. (0.17 MB DOC) [file pone.0013099.s001.doc]

**Table 1: CharacterisTable 3: Gene-profiles in PBMCs and lung aspirates**

| **Relative Quantitation (Mean±SE)** | | | | | | |
| --- | --- | --- | --- | --- | --- | --- |
|  | **PBMCs** | | | | | **LUNG ASPIRATE** |
| **GENES** | **Mild cases (n=13)** | ***p Value^a^*** | **Critically ill patients (n=11)** | ***p Value^b^*** | ***p Value^c^*** | **Critically ill patients (n=3)** |
| **Chemokines** | | | | | | |
| CCL2 | 21.49 ± 13.21 | 0.003 | 3.67 ± 1.59 | 0.757 | 0.022 | 524.24 ± 298.24 |
| CCL3 | 1.54 ± 0.83 | 0.180 | 0.05 ± 0.02 | 0.001 | 0.884 | 14.04 ± 11.31 |
| CCL5 | 1.23 ± 0.15 | 0.423 | 0.65 ± 0.20 | 0.123 | 0.022 | 0.18 ± 0.10 |
| CCL19 | 1.47 ± 0.76 | 0.423 | 0.09 ± 0.02 | 0.001 | 0.059 | 25.31 ± 19.01 |
| CCR4 | 0.6 ± 0.11 | 0.016 | 0.36 ± 0.13 | 0.031 | 0.059 | 3.48 ± 1.72 |
| CCR5 | 2.74 ± 0.55 | 0.181 | 0.87 ± 0.29 | 0.355 | 0.013 | 0.18 ± 0.10 |
| CCR7 | 0.89 ± 0.18 | 0.423 | 0.43 ± 0.12 | 0.030 | 0.052 | 0.15 ± 0.08 |
| CXCL8 | 0.91 ± 0.45 | 0.181 | 0.24 ± 0.10 | 0.355 | 0.542 | 92.86 ± 54.16 |
| CXCL10 | 3.63 ± 1.36 | 0.423 | 3.08 ± 1.32 | 0.355 | 0.192 | 51.43 ± 39.47 |
| CXCL11 | 3.82 ± 1.42 | 0.016 | 3.21 ± 1.64 | 0.355 | 0.139 | 106.12 ± 95.22 |
| **Pro-Inflammatory cytokines** | | | | | | |
| IL1A | 26.89 ± 17.95 | 0.181 | 0.28 ± 0.13 | 0.030 | 0.294 | 1,878.28 ± 1,613.63 |
| IL1B | 8 ± 4.16 | 0.423 | 0.36 ± 0.18 | 0.030 | 0.369 | 100.23 ± 81.75 |
| IL2RA | 0.74 ± 0.12 | 0.181 | 0.42 ± 0.14 | 0.030 | 0.034 | 1.88 ± 0.39 |
| IL6 | 31.02 ± 26.45 | 0.789 | 2.05 ± 0.94 | 0.003 | 0.339 | 387.92 ± 383.30 |
| IL17 | 72.54 ± 21.93 | 0.004 | 535.79 ± 324,61 | 0.005 | 0.479 | 84.79 ± 78.31 |
| TNF | 2.44 ± 0.85 | 0.789 | 0.14 ± 0.05 | 0.001 | 0.001 | 9.66 ± 7.97 |

| **Th-1 Cytokines** | | | | | | |
| --- | --- | --- | --- | --- | --- | --- |
| IFNG | 0.69 ± 0.15 | 0.061 | 0.34 ± 0.12 | 0.005 | 0.052 | 1.93 ± 1.51 |
| IL2 | 0.31 ± 0.08 | 0.001 | 0.04 ± 0.02 | 0.001 | 0.001 | ND |
| IL12A | 1.21 ± 0.16 | 0.181 | 1.01 ± 0.024 | 0.355 | 0.434 | 0.14 ± 0.05 |
| IL12B | 4.05 ± 2.39 | 0.423 | 0.61 ± 0.28 | 0.123 | 0.749 | 66.24 ± 10.76 |
| IL15 | 0.062 ± 0.14 | 0.003 | 0.73 ± 0.36 | 0.005 | 0.400 | 0.78 ± 0.47 |
| **Th-2 cytokines** | | | | | | |
| IL4 | 0.26 ± 0.06 | 0.001 | 2.66 ± 2.60 | 0.014 | 0.016 | 0.04 ± 0.02 |
| IL5 | 669.78 ± 381.88 | 0.002 | 350.21 ± 125.62 | 0.005 | 0.831 | 3.95 ± 3.16 |
| IL9 | 4.63 ± 2.36 | 0.789 | 7.73 ± 3.02 | 0.001 | 0.353 | 0.38 ± 0.24 |
| IL10 | 4.67 ± 2.48 | 0.181 | 6.95 ± 2.06 | 0.123 | 0.173 | 37.23 ± 11.55 |
| IL13 | 0.30 ± 0.10 | 0.003 | 0.06 ± 0.03 | 0.001 | 0.029 | 3.32 ± 3.24 |
| **Co-stimulatory molecules** | | | | | | |
| CD3E | 0.82 ± 0.13 | 0.061 | 0.19 ± 0.08 | 0.001 | 0.001 | 0.08 ± 0.05 |
| CD4 | 0.99 ± 0.11 | 0.789 | 0.26 ± 0.14 | 0.005 | 0.001 | 0.11 ± 0.08 |
| CD8A | 0.85 ± 0.13 | 0.423 | 0.28 ± 0.10 | 0.005 | 0.003 | 0.24 ± 0.13 |
| CD28 | 0.90 ± 0.16 | 0.789 | 0.24 ± 0.09 | 0.001 | 0.004 | 0.05 ± 0.03 |
| CD34 | 0.80 ± 0.21 | 0.181 | 0.69 ± 0.44 | 0.005 | 0.063 | 0.01 ± 0.01 |
| CD40 | 1.64 ± 0.22 | 0.181 | 0.86 ± 0.20 | 0.354 | 0.029 | 1.63 ± 0.45 |
| CD40LG | 0.99 ± 0.14 | 0.789 | 0.35 ± 0.10 | 0.001 | 0.002 | 0.04 ± 0.02 |
| CD68 | 1.22 ± 0.16 | 0.061 | 0.99 ± 0.24 | 0.757 | 0.235 | 5.14 ± 3.25 |

| CD80 | 1.67 ± 1.01 | 0.181 | 0.51 ± 0.16 | 0.030 | 0.147 | 7.55 ± 4.04 |
| --- | --- | --- | --- | --- | --- | --- |
| CD86 | 1.39 ± 0.19 | 0.061 | 0.74 ± 0.29 | 0.123 | 0.009 | 1.06 ± 0.54 |
| CTLA4 | 1.02 ± 0.19 | 0.789 | 0.18 ± 0.06 | 0.001 | 0.000 | 0.51 ± 0.23 |
| FASLG | 1.72 ± 0.28 | 0.061 | 0.57 ± 0.19 | 0.123 | 0.003 | 0.96 ± 0.60 |
| HLADRA | 1.32 ± 0.12 | 0.016 | 0.39 ± 0.15 | 0.005 | 0.001 | 0.52 ± 0.27 |
| HLADRB1 | 1.59 ± 0.92 | 0.174 | 1.92 ± 1.41 | 0.027 | 0.598 | 5,867.25 ± 5,596.77 |
| ICOS | 1.73 ± 0.28 | 0.181 | 0.54 ± 0.19 | 0.123 | 0.001 | 0.34 ± 0.14 |
| ICAM1 | 1.93 ± 0.84 | 0.423 | 0.71 ± 0.34 | 0.060 | 0.052 | 32.65 ± 14.81 |
| **Other Genes** | | | | | | |
| ACE | 0.79 ± 0.17 | 0.181 | 0.24 ± 0.10 | 0.005 | 0.002 | 5.59 ± 5.12 |
| BAX | 1.01 ±0.09 | 0.789 | 0.71 ± 0.15 | 0.030 | 0.052 | 1.25 ± 0.40 |
| BCL2 | 0.97 ± 0.21 | 0.789 | 0.37 ± 0.16 | 0.005 | 0.029 | 0.18 ± 0.12 |
| C3 | 1.31 ± 0.30 | 0.789 | 0.49 ± 0.21 | 0.030 | 0.009 | 98.98 ± 38.14 |
| COL4A5 | 0.65 ± 0.22 | 0.181 | 0.55 ± 0.32 | 0.005 | 0.369 | 1,337.78 ± 696.79 |
| CSF1 | 0.92 ± 0.39 | 0.061 | 1.52 ± 1.08 | 0.354 | 0.339 | 35.72 ± 7.59 |
| CSF2 | 1.47 ± 0.57 | 0.789 | 0.04 ± 0.02 | 0.001 | 0.000 | 6.71 ± 5.96 |
| CSF3 | 16.68 ± 15.38 | 0.180 | 25.66 ± 18.63 | 0.419 | 0.661 | 76,829.62 ± 46,813.17 |
| CYP7A1 | 18.85 ± 8.66 | 0.009 | 89.70 ± 60.67 | 0.003 | 0.354 | 77.10 ± 36.86 |
| EDN1 | 2.54 ± 1.23 | 0.789 | 0.76 ± 0.42 | 0.030 | 0.067 | 113.81 ± 92.87 |
| FN1 | 8.73 ± 7.61 | 0.181 | 5.13 ± 1.74 | 0.355 | 0.235 | 135.43 ± 36.18 |
| GNLY | 1.47 ± 0.19 | 0.061 | 0.46 ± 0.15 | 0.005 | 0.001 | 0.35 ± 0.49 |
| GZMB | 1.50 ± 0.28 | 0.423 | 0.73 ± 0.21 | 0.123 | 0.029 | 1.17 ± 0.57 |

| HMOX1 | 1.40 ± 0.23 | 0.789 | 1.41 ± 0.60 | 0.355 | 0.111 | 23.45 ± 7.14 |
| --- | --- | --- | --- | --- | --- | --- |
| IKBKB | 0.73 ± 0.11 | 0.181 | 0.42 ± 0.13 | 0.030 | 0.045 | 0.56 ± 0.15 |
| LTA | 2 ± 0.27 | 0.061 | 0.83 ± 0.24 | 0.123 | 0.002 | 0.80 ±0.49 |
| NFKB2 | 1.03 ± 0.21 | 0.423 | 0.64 ± 0.21 | 0.123 | 0.173 | 7.41 ± 2.54 |
| NOS2A | 0.33 ± 0.14 | 0.003 | 0.20 ± 0.09 | 0.005 | 0.523 | 224.02 ± 198.72 |
| PRF1 | 0.97 ± 0.18 | 0.789 | 0.31 ± 0.11 | 0.005 | 0.005 | 0.29 ± 0.16 |
| PTPRC | 0.75 ± 0.12 | 0.423 | 0.50 ± 0.16 | 0.030 | 0.098 | 1.00 ± 0.32 |
| RPL3L | 49.21 ± 21.49 | 0.006 | 35.29 ± 3.33 | 0.008 | 0.563 | ND |
| SKI | 0.79 ± 0.16 | 0.789 | 0.46 ± 0.15 | 0.005 | 0.068 | 1.25 ± 0.58 |
| SELE | 18.27 ± 2.38 | 0.009 | 82.86 ± 7.70 | 0.003 | 0.354 | 186.04 ± 50.00 |
| SMAD3 | 1.31 ± 0.20 | 0.181 | 0.46 ± 0.15 | 0.030 | 0.003 | 0.66 ± 0.30 |
| SMAD7 | 0.71 ± 0.14 | 0.423 | 0.24 ± 0.08 | 0.001 | 0.020 | 0.64 ± 0.37 |
| STAT3 | 0.88 ± 0.13 | 0.422 | 1.33 ± 0.48 | 0.757 | 0.434 | 3.90 ± 2 |
| TBX21 | 1.04 ± 0.16 | 0.789 | 0.24 ± 0.10 | 0.005 | 0.000 | 0.15 ± 0.09 |
| TFRC | 1.08 ± 0.19 | 0.423 | 1.18 ± 0.39 | 0.757 | 0.505 | 8.73 ± 2.73 |
| TNFRSF18 | 0.86 ± 0.13 | 0.423 | 0.56 ± 0.15 | 0.030 | 0.124 | 1.48 ± 0.61 |
| VEGF | 1.09 ± 0.22 | 0.181 | 1.07 ± 0.30 | 0.757 | 0.839 | 20.27 ± 6.86 |

a*Comparison between Mild cases and Healthy controls.

b* Comparison between Critically ill patients cases and Healthy controls.

c* Comparison between Critically ill patients cases and Mild cases
